# Multi-modal Mapping of the Face Selective Ventral Temporal Cortex–A Group Study With Clinical Implications for ECS, ECoG, and fMRI

**DOI:** 10.3389/fnhum.2021.616591

**Published:** 2021-03-15

**Authors:** Takahiro Sanada, Christoph Kapeller, Michael Jordan, Johannes Grünwald, Takumi Mitsuhashi, Hiroshi Ogawa, Ryogo Anei, Christoph Guger

**Affiliations:** ^1^Department of Neurosurgery, Nayoro City General Hospital, Nayoro, Japan; ^2^Department of Neurosurgery, Asahikawa Medical University, Asahikawa, Japan; ^3^g.tec Medical Engineering GmbH, Schiedlberg, Austria; ^4^Guger Technologies OG, Graz, Austria; ^5^Department of Neurosurgery, Juntendo University, Tokyo, Japan; ^6^Department of Pediatrics, Children’s Hospital of Michigan, Detroit Medical Center, Wayne State University, Detroit, MI, United States; ^7^Division of Neurology, The Hospital for Sick Children, Toronto, ON, Canada

**Keywords:** functional brain mapping, face selective regions, prosopagnosia, ventral temporal cortex, electrical cortical stimulation, electrocorticography, functional magnetic resonance imagining, epilepsy surgery

## Abstract

Face recognition is impaired in patients with prosopagnosia, which may occur as a side effect of neurosurgical procedures. Face selective regions on the ventral temporal cortex have been localized with electrical cortical stimulation (ECS), electrocorticography (ECoG), and functional magnetic resonance imagining (fMRI). This is the first group study using within-patient comparisons to validate face selective regions mapping, utilizing the aforementioned modalities. Five patients underwent surgical treatment of intractable epilepsy and joined the study. Subdural grid electrodes were implanted on their ventral temporal cortices to localize seizure foci and face selective regions as part of the functional mapping protocol. Face selective regions were identified in all patients with fMRI, four patients with ECoG, and two patients with ECS. From 177 tested electrode locations in the region of interest (ROI), which is defined by the fusiform gyrus and the inferior temporal gyrus, 54 face locations were identified by at least one modality in all patients. fMRI mapping showed the highest detection rate, revealing 70.4% for face selective locations, whereas ECoG and ECS identified 64.8 and 31.5%, respectively. Thus, 28 face locations were co-localized by at least two modalities, with detection rates of 89.3% for fMRI, 85.7% for ECoG and 53.6 % for ECS. All five patients had no face recognition deficits after surgery, even though five of the face selective locations, one obtained by ECoG and the other four by fMRI, were within 10 mm to the resected volumes. Moreover, fMRI included a quite large volume artifact on the ventral temporal cortex in the ROI from the anatomical structures of the temporal base. In conclusion, ECS was not sensitive in several patients, whereas ECoG and fMRI even showed activation within 10 mm to the resected volumes. Considering the potential signal drop-out in fMRI makes ECoG the most reliable tool to identify face selective locations in this study. A multimodal approach can improve the specificity of ECoG and fMRI, while simultaneously minimizing the number of required ECS sessions. Hence, all modalities should be considered in a clinical mapping protocol entailing combined results of co-localized face selective locations.

## Introduction

Recognizing faces seems to be a simple task, which most people perform often and unconsciously in daily life. Losing the ability to recognize unfamiliar or even familiar faces–even relatives–can tremendously decrease the quality of life. This disorder is called prosopagnosia and is characterized by impaired face recognition without other problems with visual acuity (Bodamer, 1947). It often occurs in cases with bilateral lesions in the occipital temporal cortex, especially the fusiform gyrus (Damasio et al., 1982), but also due to unilateral lesions in the right occipital temporal cortex (Benton, 1990; Takahashi et al., 1995; Wada and Yamamoto, 2001) and, in rare occasions, even on the left side (Mattson et al., 2000). Such lesions can be a side effect of neurosurgical operations of epilepsy or brain tumor (Mesad et al., 2003; Barton, 2008; Corrivetti et al., 2017). Generally, functional deficits are prevented, or at least reduced, by means of neuroimaging techniques. Most such testing has focused on brain regions responsible for language and motor functions (Penfield and Boldrey, 1937; Ojemann et al., 1989; Sagar et al., 2019). Face selective regions on the brain often remain unrevealed in clinical brain mapping protocols.

Initial hemodynamic neuroimaging studies, including positron emission tomography (PET) and functional magnetic resonance imagining (fMRI), localized the face area in ventral temporal occipital cortex (Sergent et al., 1992; Haxby et al., 1994; Puce et al., 1995). Later fMRI studies found a cluster specific to face recognition located at the lateral part of the middle/posterior fusiform gyrus, the fusiform face area (FFA) (Kanwisher et al., 1997), and also identified other face responsive regions in the inferior occipital gyrus, the occipital face area (OFA) (Puce et al., 1996; Gauthier et al., 2000), and superior temporal sulcus (STS) (Puce et al., 1998; Hoffman and Haxby, 2000). These three face selective regions are the core system for face perception that is processed by a distributed neural network (Haxby et al., 2000). While the STS processes changeable aspects, such as facial expressions and eye gaze, the FFA processes invariant face aspects, which is important for recognition. The OFA plays an important role in the perception of face parts and interacts with both FFA and STS (Haxby et al., 2000; Rossion et al., 2003; Ishai, 2008). Therefore, FFA and OFA play an important role for identification of faces, and, in case of a lesion, could cause prosopagnosia and related symptoms. Another hemodynamic imaging marker for visual perception can be obtained by PET, which requires the injection of radioactive markers for mapping (Haxby et al., 1994).

Electrophysiological signals, such as electrocorticography (ECoG), are a direct marker for neural activity. Subdural recordings from the human cortex showed face specific event related potentials (ERP) as an N200 potential in the fusiform gyrus (Allison et al.,1994a,b). Additional responses to presented faces include low frequency oscillations (Klopp et al., 1999), as well as broadband gamma activity (Lachaux et al., 2005; Schalk et al., 2017), which have been shown to be related to face N200 potentials (Engell and McCarthy, 2011; Ghuman et al., 2014). Analysis of temporal dynamics during visual perception have demonstrated that the FFA plays an important role in multiple face processing stages and that broadband gamma activity decay predicts the reaction time of percepts (Ghuman et al., 2014). Face selective broadband gamma activity also correlates with the hemodynamic response (Koch et al., 2009; Ojemann et al., 2010; Scheeringa et al., 2011; Hermes et al., 2012), and there is a good correspondence between ECoG potentials and fMRI face selective responses in posterior ventral temporal cortex (Puce et al., 1997; Jacques et al., 2016).

Electrical cortical stimulation (ECS) of face selective areas can induce inability to name familiar faces (Allison et al.,1994a,b), impairment of face discrimination (Mundel et al., 2003), or face categorization (Chong et al., 2013), illusory face responses (Parvizi et al., 2012; Rangarajan et al., 2014; Schalk et al., 2017), or transient prosopagnosia (Jonas et al., 2012, 2015). Like ECS, transcranial magnetic stimulation (TMS) has been demonstrated to disrupt face percepts (Pitcher et al., 2007). However, TMS mapping suffers from subject dependent protocols, especially for language mapping (Sollmann et al., 2018) and memory lateralization (Theodore, 2003), and requires a navigation system for localization (Romero et al., 2011).

All aforementioned imaging techniques have use cases in clinical practice. Especially, the non-invasive techniques can be relatively easily conducted for clinical investigations prior to brain surgeries. In case of epilepsy surgery, which this paper focuses on, invasive techniques can be considered as well as it often requires a two-stage surgery with intracranial electrodes for neuromonitoring. Hence, the most clinically relevant mapping techniques for epilepsy surgery are mainly ECS, fMRI, and ECoG.

Although ECS is the gold standard method for motor and language mapping (Ojemann et al., 1989), it is not necessarily the best method to localize sensory functions as visual perception or face recognition for two reasons. First, it is highly subjective and requires the ability of the patient to express symptoms. Second, it requires a time-consuming stimulation protocol (Wen et al., 2017). Hence, ECoG and fMRI mapping have been gaining attention as alternatives, especially to identify face selective areas. This raises the question which of the aforementioned modalities should be part of clinical mapping procedures to support surgical decision making. Mapping results obtained in a single case study demonstrated spatial overlap of two distinct human fusiform face selective locations between fMRI and ECoG in a patient whose face perception was distorted while electrically stimulating those locations (Parvizi et al., 2012). Another case study showed that electrical stimulation of face selective ERP and gamma band locations on the anterior fusiform gyrus induced transient prosopagnosia. However, fMRI could not localize these regions because of a severe signal dropout by an artifact (Jonas et al., 2015).

For a clinically relevant mapping protocol, these findings should be explored in more patients. This study validates mapping of face selective locations in fMRI, ECoG, and ECS in a group study using within-patient comparisons, to better understand their clinical relevance and to reduce post-operative prosopagnosia. As for epilepsy surgery the occipital lobe is relatively uncommon in daily clinical situations (Taylor et al., 2003; Pfaender et al., 2004), the region of interest (ROI) focuses on the ventral temporal cortex, specifically the fusiform gyrus and the inferior temporal gyrus.

## Materials and Methods

### Patients

Five consecutive patients at the Asahikawa Medical University Hospital underwent surgical treatment of intractable epilepsy between 2015 and 2018. All patients in this study required implantation of subdural electrodes followed by curative surgical treatment. They are four males and a female, whose age ranged from 17 to 37 years, and whose WAIS-III (Wechsler adult intelligence scale) full IQ was 58–105. Their pre-surgical assessment required the implantation of subdural grid electrodes on the ventral temporal cortex for diagnostic purposes, including video EEG monitoring to localize seizure onset zones and routine functional mapping. Additionally, the patients underwent an extensive ECS procedure to localize face selective locations and thus avoid post-operative deficits like prosopagnosia. The two additional functional mapping procedures in this study, ECoG and fMRI, provided supportive information. Wada test was performed in determining language lateralization. Table 1 presents the patients’ characteristics. All patients were defined as “unknown” etiology based on The International League Against Epilepsy Classification of the Epilepsies (Scheffer et al., 2017). The study was approved by the Institutional Review Board of the Asahikawa Medical University (Asahikawa Medical University Research Ethics Committee). Written informed consent, including detailed explanation, was obtained from each patient and their family.

**TABLE 1:** Demographic data for all five patients.

Patient	Age	WAIS-III Full IQ	Handedness	Language dominance	MRI findings	Seizure onset zone	OP	Symptoms	Etiology	ROI electrodes, n	Total electrodes, n	fMRI pre/post OP
1	26	96	R	L	None	R Temporal	R anterior temporal lobectomy	CPS	Unknown	R 22 L 27	R 56 L 132	Post
2	17	105	L	L	None	L Temporal	L lateral temporal lobectomy	CPS, GCS	Unknown	L 27	L 166	Post
3	22	63	R	L	None	R Temporal	R hippocampectomy	CPS, GCS	Unknown	R 37	L 160	Pre
4	23	78	R	L	None	R Temporal	R hippocampectomy	CPS, GCS	Unknown	R 40	R 160	Pre
5	37	58	R	L	None	R Temporal	R anterior temporal lobectomy	CPS, GCS	Unknown	R 25	R 102	Pre

*R, right; L, left; OP, Operation; CPS, complex partial seizures; GCS, generalized convulsive status; ROI, region of interest; WAIS-III, Wechsler Adult Intelligence Scale-III.*

**FIGURE 1:**
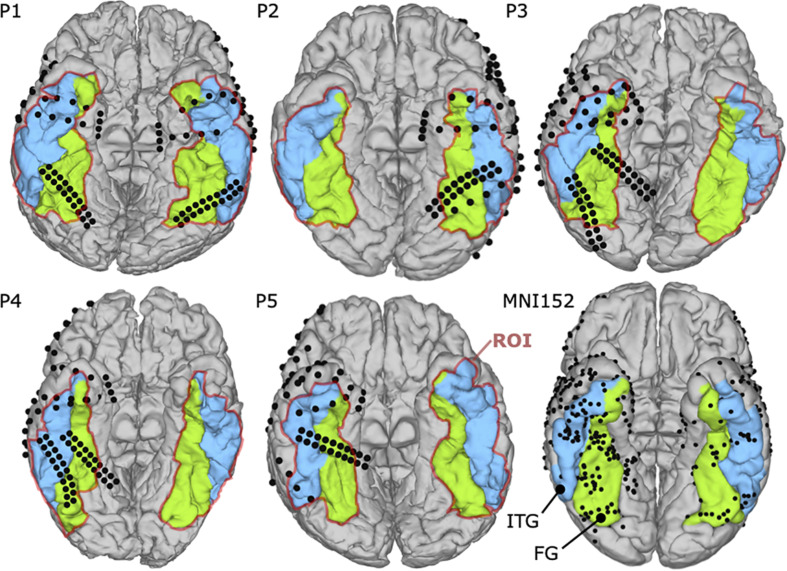
Locations of the implanted electrodes (black balls) on the MNI152 brain and the individual coverage of each patient. The selected region of interest (ROI) is defined by colored cortex regions, including the fusiform gyrus (FG) (green colored) and the Inferior temporal gyrus (ITG) (blue colored).

Each patient had a pre-operative computerized tomography (CT) scan to identify electrode locations in conjunction with pre-operative magnetic resonance imaging (MRI) as part of the fMRI mapping. Each patient’s brain was reconstructed in FreeSurfer (Martinos Center for Biomedical Imaging, Cambridge, MA, United States) using the T1-weighted MRI (Dale et al., 1999). Then, pre-operative MRI data were co-registered to post-operative CT scans using SPM12 (Wellcome Centre for Human Neuroimaging, London, United Kingdom) to localize electrode positions on the cortex (Penny et al., 2011). Figure 1 shows the projected electrode locations of all patients on the MNI152 (Montreal Neurological Institute) brain.

### Mapping Procedures

#### fMRI

**FIGURE 2:**
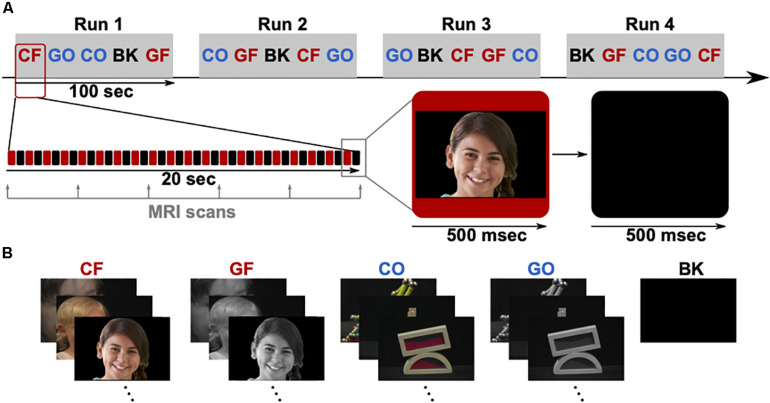
**(A)** fMRI paradigm consisting of four runs (Run 1–4), each containing five blocks in pseudo-randomized order. The five blocks are colored faces (CF), grayscale faces (GF), colored objects (CO), grayscale objects (GO), and black screens (BK). Each block lasts 20 s, showing 20 stimuli (500 ms presentation time). **(B)** Example stimuli for each block. The visible face is representative for the original stimuli and used from a public database (copyright shutterstock).

The fMRI examinations were performed with a 3.0 T whole-body MR scanner with echo-planar capabilities and 32-channel surface coil (Discovery 750 W; General Electric, Milwaukee, WI, United States). All patients participated in the fMRI mapping, three of them pre-operatively (P3, P4, and P5) and two post-operatively (P1 and P2). Specifically, fMRI for P1 and P2 were obtained 3 and 1 year after surgery, respectively. During the scans, foam cushions stabilized their heads to prevent motion artifacts. First, a high-resolution T1-weighted 3D volumetric scan was obtained for co-registration of the functional maps, consisting of 1.2 mm thick axial slices with a resolution of 256 × 256 pixels in a field of view of 240 mm. As depicted in Figure 2, five task block conditions were acquired during fMRI to reveal functional brain regions specific to visual categorization. These conditions included photographs of faces and objects, either colored or grayscale, and black screens. Patients were asked to look at a 32′ monitor about 270 cm away, yielding a visual angle of 14.9° × 8.3°. They focused on the visual paradigm while they were shown visual stimuli (faces, objects or black screens) for 500 ms and subsequent 500 ms black screen over a period of 100 s in total. The relative luminance (Rec. ITU-R BT.709-6) of 0.058 for faces and 0.046 for objects was similar in both conditions, and the medium spatial frequency band of 5–15 cycles/face, which is most relevant for face processing (Collin et al., 2012), consisted of 13.2% or −8.79 dB of the whole signal energy for both, faces and objects.

The presenter in the g.HIsys online processing toolbox in Simulink (g.tec medical engineering GmbH, Austria) managed the paradigm and stimuli. Stimulus presentation was synchronized with the scanner using TTL trigger. During the presentation period, a T2-weighted echo-planar imaging sequence acquired dynamic volumes of the task block conditions (TE = 30 ms; TR = 4000 ms; flip angel: 80°; slice thickness: 3 mm; field of view: 240 mm; matrix: 64 × 64; number of 40 slices). Each block contained five echo-planar imaging volumes. The experiment was repeated four times in pseudo-randomized order, yielding 25 volumes per sequence and three additional preceding dummy scans. At the end of the fMRI mapping, 40 volumes per condition (face and object) were available for statistical analysis in SPM12 (Wellcome Centre for Human Neuroimaging, United Kingdom). Parameters were mainly selected based on the suggested analysis pipeline for the high-level visual cortex (Weiner and Grill-Spector, 2013) and previously used settings to obtain faces selective cortex regions on the ventral temporal cortex (Winston et al., 2004; Liu et al., 2008; Jonas et al., 2015; Schwarz et al., 2019; Farmer et al., 2020).

The fMRI scans were pre-processed including: (a) realignment of the fMRI time-series, (b) co-registration of the realigned functional images on the anatomical MRI volume by maximizing their normalized mutual information with the mean functional image, and (c) spatial smoothing with an isotropic Gaussian kernel (2 mm). For the analysis, a first-level model was specified and estimated, data were corrected for low frequency drifts (128 s high pass filter) and corrected for serial correlations with a first-order autoregressive model. A binary mask of gray matter was used to constrict the analysis. The mask was created by segmenting the structural MRI volume in SPM12. We concatenated all runs by replacing the usual mean column in the design matrix with regressors modeling each session and adjusting the high-pass filter and non-sphericity calculations as if sessions were separate. For each patient, a t-map volume was created using the contrast of interest (i.e., presentation times of gray and colored faces against those of gray and colored objects).

The SPM t-map volumes were mapped onto the surface of the corresponding reconstructed brains (triangulated meshes). The *t*-values were assigned to the surface vertices by averaging the voxel intensities along 6 mm of the vertex normal directions using Gaussian weights (FWHM = 10 mm) (Hermes et al., 2012; Gaglianese et al., 2017; Haufe et al., 2018). For each electrode location, a *t*-value was determined by averaging the highest 5% of the *t*-values within a radius of 6 mm (Hermes et al., 2012; Gaglianese et al., 2017; Haufe et al., 2018). Electrodes with a *t*-value > 3.3 were considered as fMRI positive, which corresponds to a statistically significant activation threshold of *p* < 0.001 (uncorrected, DOF = 87).

#### ECoG

Intracranial EEG was obtained from subdural grid electrodes (Unique Medical Co., Ltd., Japan) with 1.5–3.0 mm exposed diameter and 5–10 mm spacing, which were originally implanted for epilepsy monitoring. Data were recorded at the bedside by a DC coupled g.HIamp biosignal amplifier (g.tec medical engineering GmbH, Austria) and digitized with 24-bit resolution at a sampling rate of at least 1200 Hz. Dorsal parietal electrodes served as ground (GND) to ensure no overlap with the ROI.

**FIGURE 3:**
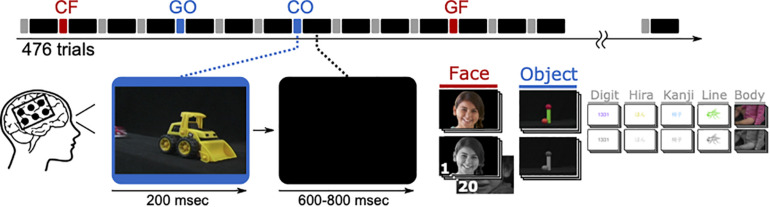
One ECoG paradigm lasts 476 trials, each with 200 ms presentation time and 600–800 ms inter-stimulus-interval, resulting in 333 s on average. Trials showing faces (CF and GF) or objects (CO and GO) were considered in the analysis. The visible face is representative for the original stimuli and used from a public database (copyright shutterstock).

A 14′ presentation monitor was placed at 80 cm in front of the patient’s face, showing visual stimuli to the patients, who were instructed to look at the monitor and focus on the paradigm in Figure 3. The resultant visual angle was 22.0° × 12.5°. The visual stimulation paradigm contained seven different types in colored and grayscale variants. Among others, photographs of faces and objects were displayed for 200 ms and followed by black screen for 600–800 ms. Each type was presented 68 times, randomly chosen out of a pool of 20 different pictures. Presented images of faces and objects were identical with those during fMRI.

Electrocorticography data were acquired and processed by means of the g.HIsys real-time processing library (g.tec medical engineering, Austria), which also controlled the experimental paradigm and stimulus presentation. Initial processing steps included a remove drift filter (2 Hz high-pass, fourth order Butterworth) and a common average reference. Afterward, a combination of band-pass filter (110–140 Hz, Butterworth, low and high pass of fourth order each) and Hilbert transform resulted in broadband gamma signals, which were down-sampled (to 400 Hz) and square-root transformed to approximate Gaussianity. Finally, signals were standardized with respect to 300 ms periods immediately preceding stimulus presentation (−300 to 0 ms).

Changes in broadband high gamma activity of the active face phases (100–400 ms: post-stimulus period) compared with active object phases (100–400 ms: post-stimulus period) were calculated into coefficient of determination (*r*^2^) for each individual electrode in each paradigm. The *r*^2^ value can be translated to a *t*-value under consideration of the sample size (i.e., number of trials denoted by N_OBJ and N_FACE) as follows:

$$t=\sqrt{\frac{r^{2}\,\left(N_{\text{OBJ}}+N_{\text{FACE}}-2\right)}{1-r^{2}}}$$

Thus, an *r*^2^ > 0.02 yields *t* > 1.98 (*p* < 0.05, two-tailed), and after Bonferroni correction for up to 188 electrode locations per patient, a critical *r*^2^ > 0.10 (*t* > 3.74) reliably indicates significant difference of object and face related high gamma activity. Hence, ECoG locations were considered as face selective if they exceeded *r*^2^ > 0.1.

#### ECS

Functional mapping through ECS was performed at the same locations as the ECoG mapping. Electrical stimulation trains of 3–10 s were injected into pairs of adjacent electrodes on the patient’s brain surface using biphasic constant current pulses (50 Hz train rate; 0.2 ms pulse duration) using Neuromaster MEE-1232 (Nihon Kohden Co., Japan). Current amplitudes started from 3 mA and increased progressively until symptoms occurred, or up to 10 mA at maximum. In an initial motor mapping, the minimum current threshold necessary to elicit movement was determined in each patient. Then, stimulation was applied in the same way to map the temporal-occipital face recognition. During the stimulation, the patient sat and leaned back on the bed to relax. The patient was shown human face photographs and asked to report any change in perception. If a patient perceived any change or felt something while looking at the photographs, the patient was further asked to look at objects and the presenter’s own faces to discriminate face related from object related responses. A region was defined as ECS positive (or face specific) if a patient reported consistent face illusions (or any consistent change in the face perception) that did not occur in objects while looking at faces and objects.

### Surgical Outcome Evaluation

Post-operative T1-weighted MRI was obtained from each patient to extract the resected volumes. The electrode locations within 10 mm distance to the resected volume were defined as resected locations to comply with the 10 mm safety margin published in previous mapping studies (Haglund et al., 1994; Sanai et al., 2008; Swift et al., 2018).

## Results

### ECS

**FIGURE 4:**
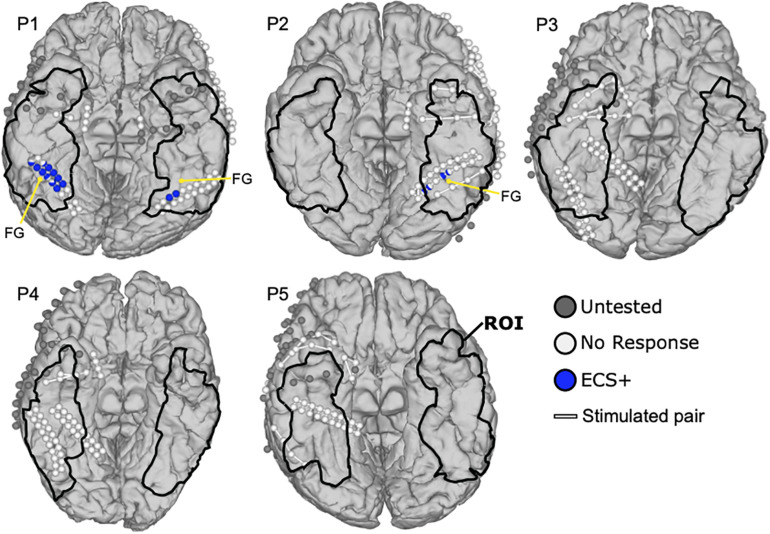
Mapping results of ECS for face perception. Blue colored balls represent face selective locations during ECS that caused reported symptoms in the patients. White balls are silent locations without reported symptoms and gray ones were not stimulated because of limited mapping time. White lines connect the electrode pairs that were stimulated together. The region of interest (ROI) is marked with black lines. Anatomical landmarks show the fusiform gyrus (FG).

Figure 4 shows the ECS results of patients P1 to P5. Two of five patients (P1, P2) reported face related symptoms in response to ECS in 17 locations within the ROI. On the other hand, 21 electrodes in ROI were not tested by ECS because of the electrode issues, time effort or burden for the patient, or the high risk of inducing a seizure near the seizure onset zone. Patient P1 reported that the face and eyes completely changed during stimulation while he observed a real face. He further mentioned seeing an eye and mouth on the box, the ball and the kanji and their shapes did not change. Thus, two functional clusters were revealed covering 13 ECS positive locations in blue, one on the right and the other one on the left lateral fusiform gyrus. Notably, the cluster on the right hemisphere (11 locations) was larger than the one on the left (two locations), but their locations were almost symmetric. Also, the four face specific locations in P2 could be clustered in two regions of the left fusiform gyrus, two at the medial side and the other two at the lateral side. During the stimulation, P2 experienced different sized right and left halves of the observed face, and reported that the right and left halves of the faces were from different people, which P2 described as “Picasso pictures.” This ECS symptom occurred only while looking at faces, but not for objects. The remaining cases did not report any face specific symptoms elicited by ECS.

### ECoG

**FIGURE 5:**
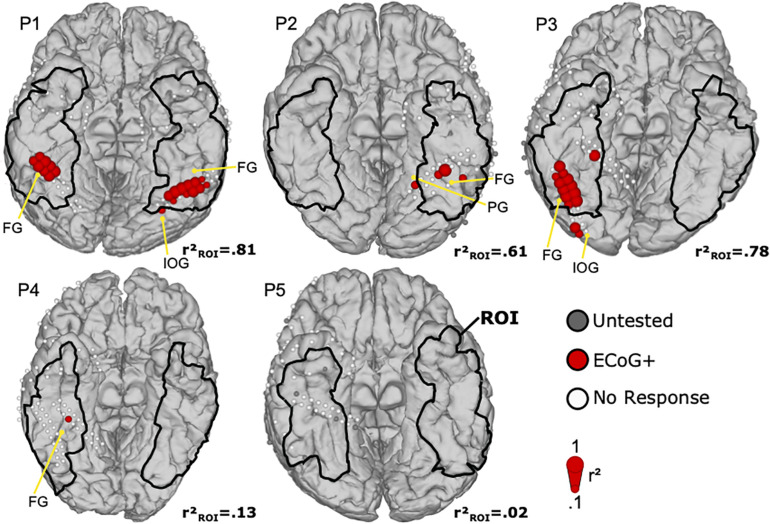
Mapping results of ECoG for face perception. Face selective ECoG locations are denoted by filled red circles. The diameter of each circle corresponds to the face related *r*^2^ value of that location. White ECoG locations did not exceed the significance threshold, and gray locations had to be excluded from further processing due to bad signal quality after visual inspection. The region of interest (ROI) is marked with black lines. Anatomical landmarks show the fusiform gyrus (FG), the inferior occipital gyrus (IOG), and the parahippocampal gyrus (PG).

Figure 5 shows the ECoG mapping results with face selective locations in the ROI. In total, 35 face locations were identified in four of five patients (P1–P4). Four electrodes in ROI were not tested by ECoG bad signal quality due to electrode attachment. In P1, two bilateral activation clusters of 8 (right) and 11 (left) locations were found at the middle lateral side of fusiform gyrus, with almost symmetric activation patterns. One location in the left inferior occipital gyrus was excluded as it was outside the ROI. P2 demonstrated three face selective locations in the ROI at the lateral side of fusiform gyrus. Another face selective location was in the parahippocampal gyrus, outside the ROI, and thus excluded. In P3, 11 face selective ECoG locations were assembled in a large cluster at the right lateral side of fusiform gyrus. Another two ECoG positive locations were at the occipital lobe and out of ROI. Finally, one face selective site was found in P4 and located on the right middle lateral side of fusiform gyrus, consistent with the expected face area. P5 had no ECoG positive locations, but showed high impedance in four ECoG locations, and hence these areas were not tested during the ECoG mapping.

### fMRI

**FIGURE 6:**
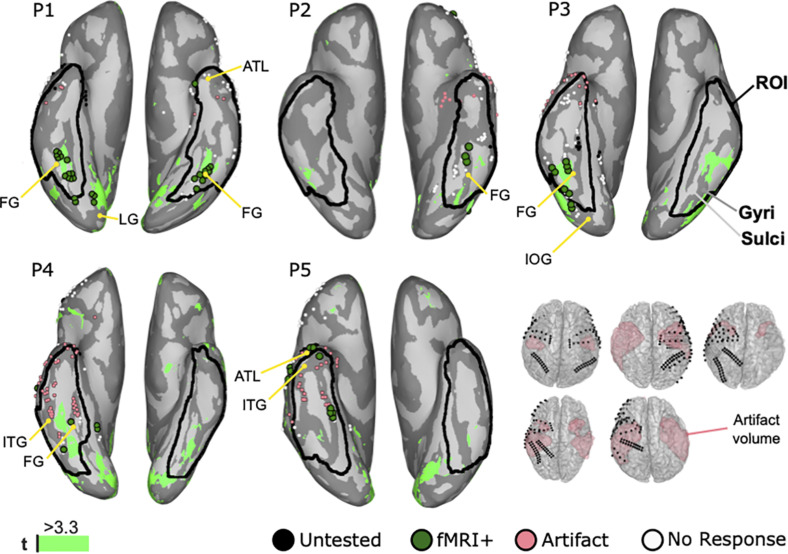
Mapping results of fMRI for face perception on inflated brains of five patients with highlighted region of interest (ROI). Inflated brains illustrate gyri (dark gray) and sulci (light gray) to reveal hidden activation. Anatomical landmarks show the fusiform gyrus (FG), the inferior temporal gyrus (ITG), the lingual gyrus (LG), the inferior occipital gyrus (IOG), and the anterior temporal lobe (ATL). White dots represent electrodes’ locations on the gyri. Significantly higher BOLD responses to faces compared to objects are colored in green (light green: *t* ≥ 3.3). Black dots are untested locations due to post-surgical fMRI. Coral dots are locations contaminated by the MRI artifacts shown the volume plots.

Figure 6 shows the fMRI mapping results from all cases. In all cases, the BOLD signals responded significantly higher to faces than objects in middle lateral regions of the fusiform gyrus in either one or both hemispheres. Those 38 face selective locations that were lying within 6 mm of ECoG locations were further denoted as fMRI positive locations and occurred in all patients. Artifacts in the fMRI caused a signal drop-out in 60 electrodes locations in the ROI. In P1, 21 fMRI positive (fMRI+) locations were detected in the ROI of both hemispheres. Thus, clusters of twelve in the right and nine in the left side were symmetrically located at the fusiform gyrus. Three locations in the right lingual gyrus, two in the right inferior occipital gyrus, and one in the left anterior temporal lobe were outside the ROI and thus rejected. In P2, the cluster of three fMRI positive locations is in the left fusiform gyrus. P3 demonstrated seven fMRI positive locations in the lateral side of the right fusiform gyrus. Another two posterior locations were out of the ROI in the inferior occipital gyrus and thus excluded. In P4, two locations are in the right fusiform gyrus. On the other hand, two locations in the right lingual gyrus were excluded. In P5, one electrode was at the anterior rim of inferior temporal gyrus and four electrodes in the fusiform gyrus. Two locations in the anterior temporal lobe were out of the ROI. In addition to the aforementioned face selective fMRI locations, more regions were identified in the frontal lobe, the lateral temporal lobe, the parietal lobe, and the occipital lobe, but were rejected because those regions were outside the ROI.

### Comparison Among Three Modalities

**FIGURE 7:**
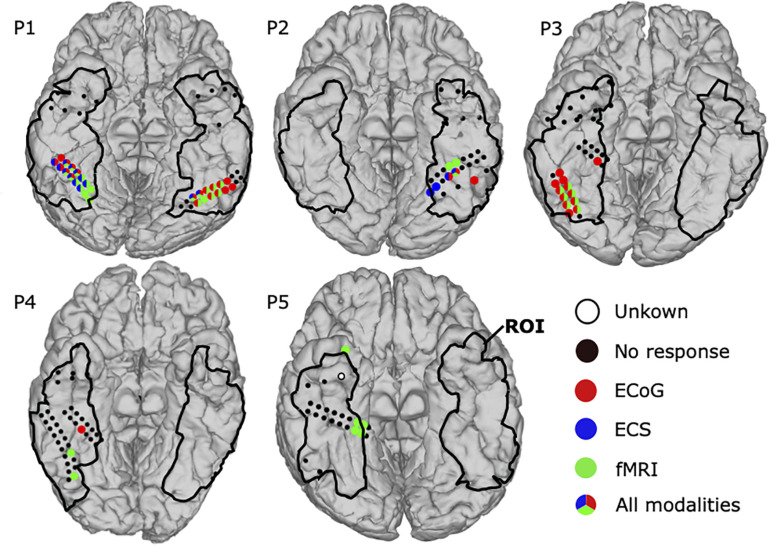
Mapping results for comparison among three modalities: ECoG, ECS, and fMRI. Face selective locations in the ROI are shown as filled circles, detected by ECoG (red), ECS (blue), and fMRI (green). Locations identified by multiple modalities have circles with mixed colors. Black dots represent locations without any face related response. The region of interest (ROI) is marked with black lines. Untested locations are highlighted in white.

Figure 7 shows the combined results for ECoG, ECS, and fMRI. In total 178 locations were in the ROI and 54 face locations were identified by at least one modality in all patients. Detection rates were 70.4, 64.8, and 31.5% for fMRI, ECoG, and ECS, respectively. Thus, 28 of those areas were co-localized by at least two modalities, with detection rates of 89.3% for fMRI, 85.7% for ECoG, and 53.6% for ECS. Eight face locations were co-localized in all three modalities in P1 and P2. In P1, All of them covered the bilateral lateral fusiform gyrus in two small clusters, five adjacent locations on the right and two adjacent locations on the left hemisphere. The clusters were surrounded by face selective locations identified by two modalities (mixed paired combinations of ECoG, ECS, and fMRI). ECS biased toward the right one and both fMRI and ECoG equally distributed on both sides. Finally, four ECoG and four fMRI positive locations remained unconfirmed by another modality. P2 also reported face locations by all three modalities. One location in fusiform gyrus was co-localized in all three modalities and one location medial side to it was commonly activated in the lateral fusiform gyrus in both ECS and ECoG. On the other hand, two locations were identified by fMRI anterior to the co-localized face locations and one more lateral location was identified by ECoG only. Those face locations clustered in left fusiform gyrus.

Another two patients responded with face selective locations in at least two modalities. In P3, fMRI and ECoG mappings revealed 7 and 12 face selective locations, respectively. All seven fMRI positive locations were located in the right fusiform gyrus, co-activated in ECoG, and surrounded by the remaining five ECoG positive locations. In P4, ECoG mapping detected one positive location and fMRI found two positive locations, but no location was co-localized.

One patient, P5, showed activation in only a single modality. Thus, fMRI detected five positive locations, one at inferior temporal lobe and the other four at right medical fusiform gyrus within the ROI. P5 further had one “unknown electrode,” because it was not tested by ECS and ECoG, and also affected by the fMRI artifact (closer than 6 mm).

### Extent of Resection, Functional Outcome and Related Electrode Locations

**FIGURE 8:**
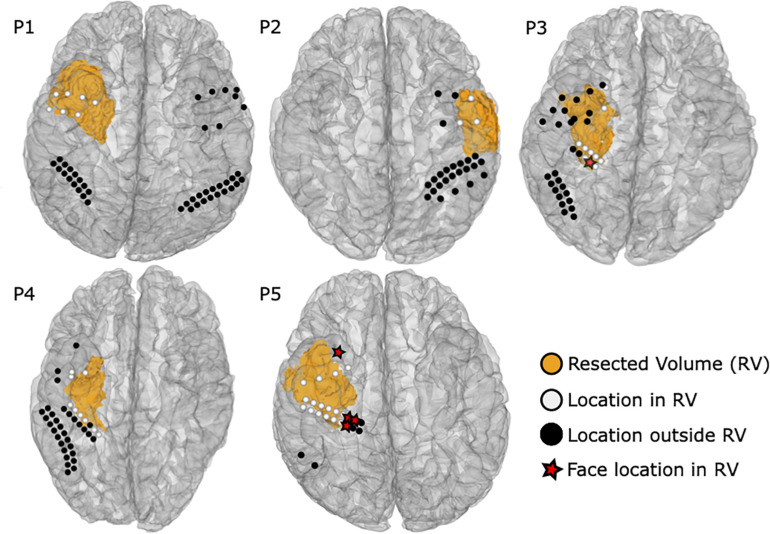
Resected volumes from the post-operative MRI are shown as orange blobs. ECoG and ECS locations are colored white within 10 mm from the resected volume, whereas black labeled ROI locations were considered as distant and thus not relevant to the surgical outcome. Red stars highlight face selective locations in the resected volume.

Figure 8 shows the extent of resection together with 49 ECoG and ECS locations near the resected volumes. Five resected locations were identified as face selective, one by ECoG in P3 and the other four by fMRI in P5. None of the patients suffered from prosopagnosia, as each of them could recognize their doctors’, nurses’, and family faces after surgery. Hence, the five locations were considered as false positive. One location in P5 remained untested, yielding 43 locations which were tested by at least one modality near the resected area were considered as true negative.

Eleven ECS, three ECoG, and 33 fMRI locations could not be tested during the individual sessions, but got tested by at least another modality. Six electrodes inside the resection area in P1 were not stimulated because of after discharges and the high risk of inducing a seizure near the seizure onset zone. One resected location could not be stimulated in P3 due to bad electrode attachment. The last four not stimulated locations were located in P5, which included a broken electrode connection. During fMRI, two electrodes in P1 and one electrode in P2 inside the resection area were not tested by fMRI, as the scans were obtained post-operatively. Moreover, the number of locations in the resection area that were contaminated by an fMRI artifact was 3, 1, 2, 8, and 16 in P1 to P5, respectively. The four untested ECoG locations were excluded because of bad signal quality due to electrode attachment.

## Discussion

Face selective cortex regions have been revealed in numerous neuroimaging studies based on ECS, ECoG, or fMRI. Most of them either utilized only one modality or demonstrated findings in individual subjects. To the best of our knowledge, this is the first report on face functional mapping compared with surgical outcomes.

Although no face recognition deficits occurred after surgery, surprisingly, ECS elicited face processing symptoms in only 2/5 patients, revealing only 31.5% of all identified face selective locations. ECS did not elicit any face specific symptoms in P3, P4, and P5, while positive locations in P2 were similar to corresponding areas in the left sided cluster in P1. To date, ECS is still the gold standard method for functional brain mapping of motor and language areas (Penfield and Boldrey, 1937; Ojemann et al., 1989). However, the fact that only two out of five patients reported face selective symptoms raises doubts that ECS is robust enough to localize face perception in the brain. Previously, several studies have shown that electrocortical stimulation causes distortion of face perception or transient prosopagnosia, but the majority investigated single cases (Mundel et al., 2003; Parvizi et al., 2012; Schalk et al., 2017). In this group of five consecutive patients it seems that the inter-patient variability of the symptoms is quite high. As the ECS symptoms can be considered as transient apperceptive prosopagnosia it may be that the low sensitivity can be explained by the complex nature of the visual categorization network. The observed low sensitivity of ECS could be due to the inactivation of only parts of this network. This effect has been demonstrated in cases with prosopagnosia before, in whom it was more likely to occur in the right occipito-temporal areas, often with combined multiple lesions, including the lingual, fusiform and anterior inferior temporal cortices (Meadows, 1974; Damasio et al., 1982; Barton et al., 2002; Bukach et al., 2006; Sorger et al., 2007). Therefore, ECS may struggle to inhibit the network, since it affects a small area of the brain surface through a stimulated electrode pair.

Broadband gamma ECoG activity seems to be a more robust marker to reveal face selective locations (Jonas et al., 2016), and significantly responded only to faces in four out of five cases in this study. ECoG mapping showed the second highest detection rate, revealing 64.8% or 35 of 54 face selective locations in 4/5 patients. Thus, 85.7% or 24 of 28 face selective locations were co-localized with ECoG. This indicates that broadband gamma activity in ECoG provides higher sensitivity compared to ECS. Interestingly, in P1, all three modalities successfully detected the face selective area at fusiform gyrus and also in P2 at left fusiform gyrus similar to a previous report (Schalk et al., 2017). Notably, ECoG revealed the locations that were ECS negative in P3 and P4, but located at the middle and posterior part of the fusiform gyrus, known as the FFA (Kanwisher et al., 1997). This could be due to the subjective reporting and evaluating or ECS symptoms. ECS has major problems when distinguishing face selective from visual processing locations, and a protocol that addresses any specific visual category would be time-consuming and demanding for the patient. ECoG clearly reveals face selective locations objectively that cannot be found by ECS, either because of insensitivity of the patient or difficulty classifying the reported symptoms. This suggests that ECoG could map the FFA more effectively than ECS, without long-lasting protocols and the burden to iteratively check only a few electrodes being stimulated at a time. Taking into account the broad and complex network that is involved in face perception, ECoG, as an observational technique, may be a more practical procedure to detect the face processing network.

Face selective BOLD responses during fMRI appeared on the ventral temporal cortex in all patients, exceeding the significance threshold *t* > 3.3. In P1, P2, and P3, the activated regions overlapped with electrode locations on the lateral fusiform gyrus and were co-identified in ECoG, containing 25 out of 28 locations found by at least these two modalities. Those results support the sustained and strong correlation between fMRI and ECoG signals in the high frequency broadband range of 30–160 Hz (Jacques et al., 2016). Furthermore, in P5, fMRI was only one modality that identified four face locations at fusiform gyrus and one positive face region at anterior tip of collateral sulcus (Nasr and Tootell, 2012; Tanji et al., 2012; Jonas et al., 2016). This area was not covered by subdural electrodes. Notably, P4 showed significant fMRI activation in the sulcus between fusiform and inferior temporal gyrus, which was not visible in ECoG or ECS locations on the gyrus above. Consequently, fMRI is useful for revealing functional regions that are not covered by electrodes, and may be considered for pre-surgical mapping. This is important for surgical planning, because exact positioning of the electrodes at the temporal base can be difficult due to bridging veins, and the number of grids that can be implanted is limited. Furthermore, potential infections after implantation are a serious risk.

None of the brain surgeries required resecting ECS positive cortex locations. Notably, P1 had six locations inside the resected area, which were not stimulated because of frequent after-discharges near to the seizure onset zone and high risk of seizure. On the other hand, five face selective locations, one obtained by ECoG and another four by fMRI, were within 10 mm of the resected volumes. Thus, ECoG and, most of all, fMRI tend to be too sensitive, which is a known issue of observational mapping techniques (Tharin and Golby, 2007). Interestingly, the false positives remained unconfirmed by any other modality. All other face selective locations remained outside the resected volume, which of course cannot be validated with respect to functional outcome, since the resection area must be as small as possible. Meanwhile, 43 true negative and no false negative locations reliably predicted a safe operation without post-operative prosopagnosia. The lower specificity of ECoG and fMRI, caused by the false positive locations, could be overcome by combining mapping results of co-localized face selective locations.

Each modality comes with disadvantages that should be considered. ECS has side effects that include stimulation-induced pain and seizures. Stimulating the ventral temporal cortex caused facial pain in P2 and P5, facial numbness in P3, and uncomfortable face contraction in P4. Discomfort or pain during ECS may occur because of the limited space between dura mater and cortex. Thus, the current during ECS propagates to the dura mater or trigeminal nerve, which could obstruct the mapping or surgery. ECS also induced after discharges in P1, P3, P4, and P5, and caused a seizure in P5, and hence the mapping was stopped. fMRI could underestimate or fail to disclose face area because of other reasons, including susceptibility to artifact in the anterior half of ventral occipital temporal cortex arising from the ear canals (Weiner and Grill-Spector, 2010; Rossion et al., 2018) and in the inferior lateral temporal lobe due to the brain region adjacent to bone and air sinuses (Ojemann et al., 1997). Our study showed that 30 of 49 electrodes in resection volumes were contaminated by an fMRI artifact. This result suggests that fMRI is not sufficient for pre-surgical face mapping of the anterior ventral temporal cortex. On the other hand, based on the results in this study, face selective locations were found on the posterior parts of the ventral temporal cortex. Although the regions are quite close to those obtained with ECoG and ECS, the artifact volumes in Figure 6 raise concerns about the feasibility of fMRI to reveal all relevant face regions. The observed artifacts were mainly visible in anterior regions of the temporal lobe, in which lesions could cause associative prosopagnosia (Evans et al., 1995; Gainotti et al., 2003). Nevertheless, significant activation of face selective locations was identified in the ROI in all patients, mainly in regions related to apperceptive prosopagnosia (Damasio et al., 1982; De Renzi, 1986; De Renzi et al., 1991; Bouvier and Engel, 2006; Gainotti, 2013).

On the other hand, although no adverse events occurred during the ECoG mapping, four locations were excluded due to their high impedance for recordings during the ECoG procedure because of the problem with the electrodes’ attachment to brain surface. However, ECS faces this challenge too, because ECS also requires implanted electrodes. Thus, ECoG and fMRI have fewer disadvantages, which are basically conceptual limitations such as connection problems or artifacts. Therefore, ECoG and fMRI tend to be more practical procedures than ECS in terms of time effort (Wen et al., 2017), and–above all–they do not induce pain or seizures when mapping the ventral temporal cortex.

The current study extends existing literature using a multimodal approach with increased sensitivity and detection rate of face selective areas. Interestingly, this study showed that ECoG and fMRI could reveal face selective cortex regions that were not identified through ECS. Furthermore, fMRI negative results have to be handled with care, as not all tissue in the ROI can be mapped due to artifacts. It seems that ECoG results, although limited to the implanted locations, most reliably reveal face selective areas. In order to avoid prosopagnosia, it is also important to achieve a good predictor for the surgical outcome. Therefore, the high sensitivity of ECoG is not enough in clinical practice, considering the chance of false positive locations. To judge whether or not a resective surgery can be justified in terms of functional outcome it is important to co-localize face selective locations and thus, improve the specificity of the combined approach. In this study face selective locations were co-localized by more than two modalities in 3/5 patients at 28 locations. This also suggests that the multimodal approach is more sensitive for revealing face selective locations than ECS mapping alone. Mapping with ECS alone has a great specificity, as the symptoms caused by the stimulation mimic a potential prosopagnosia, but the low observed sensitivity for multiple patients may cause a lot of false negatives. A surgeon has to judge if the electrodes are truly negative, which can be achieved by adding multiple independent observations.

The present study has several limitations. First, the number of patients was limited to five. Hence, due to the high variability of the mapping results across the patients, it is possible that detection rates may change in a larger population. Nevertheless, this is the first study comparing face selective locations in multiple patients among ECS, ECoG and fMRI. Moreover, the 177 tested electrode locations in the ROI provide a large test population that justifies inferences based on the mapping results. Second, fMRI of P1 and P2 were taken after surgery. This still raises the concern of the remaining false negatives for fMRI. However, given the artifact effect, only two electrodes in P1 and one electrode in P2 were affected by post-surgical fMRI. Third, one electrode could not be tested by any modality, leaving a blind spot on that location. All other electrode locations were tested by at least one modality. However, the risk that locations may remain untested is high if only a single modality is used and the chance a location could not be tested was 11.80, 2.25, and 33.71% for ECS, ECoG, and fMRI, respectively. Testing all locations with each technique may not be possible given the aforementioned side effects, electrode issues, and patient condition. Therefore, combined results could at least ensure that the whole ROI is tested. Fourth, the anatomical T1 weighted image in P2 taken before surgery included severe artifacts which caused missing parts of both sides of temporal lobes. It was not possible to repeat the MRI scans, because P2 suffered from mild claustrophobia during the fMRI, but merging the T1 weighted images taken before surgery and after surgery enabled us to visualize the anatomy of the ventral temporal cortex. Although there are still unclear parts on anterior fusiform gyrus, there are basically no problems to know the positional relationship between electrodes with the anatomy of the ventral temporal cortex. Fifth, the magnitude of *t*-values related to the BOLD responses in fMRI varied across patients. To make comparison easier, the threshold for fMRI positive locations was set to *t* ≥ 3.3. Sixth, the fMRI acquisition protocol causes a signal drop-out on the anterior ventral temporal lobe due to the ear canal. Similar to other studies, a quite large volume artifact on the ventral temporal cortex in the ROI from the temporal bone, air sinuses, or ear canal, cannot be analyzed (Puce et al., 1995; Devlin et al., 2000; Ku et al., 2011; Golarai et al., 2017). The artifactual signal drop-out may be minimized by smaller voxel sizes to 1–2 mm, minimizing partial volume effects, but cannot be completely suppressed (Weiner and Grill-Spector, 2013). Seventh, the mapping protocols presented in this paper focus mainly on face perception areas of the brain, and thus, tend to avoid apperceptive prosopagnosia. Apperceptive prosopagnosia is more likely related to the damage of the fusiform gyrus (Meadows, 1974; Damasio et al., 1982; Bouvier and Engel, 2006), whereas a more associative variant is more likely related to anterior temporal damage (Evans et al., 1995; Gainotti et al., 2003). To avoid associative prosopagnosia an extension of the protocols testing for locations recognizing familiar faces could potentially improve the sensitivity of the mapping procedure. However, it could be difficult to establish such a protocol with ECS, which makes it less practicable in clinical practice. Finally, other fMRI studies revealed a cluster specific to face recognition located at the lateral part of inferior occipital gyrus, the OFA (Gauthier et al., 2000; Pitcher et al., 2011). This could be of interest for other brain surgeries like tumor resection. Since the current study focused on epilepsy surgery the inferior occipital gyrus was not included in the ROI (Taylor et al., 2003; Pfaender et al., 2004). Notably, in case of a one stage surgery under general anesthesia, like for occipital tumors, fMRI may be the most practical technique to prevent prosopagnosia.

To sum up, this is the first study for mapping of face selective locations among multimodalities, in ECS, ECoG, and fMRI, in a group study compared with surgical outcomes. Notably, ECS was least sensitive, whereas ECoG and fMRI tended to be too sensitive, as they had the face selective locations within 10 mm to the resected volumes even though the patients had no face recognition deficits. Each modality has its strengths and weaknesses. ECS is more invasive, time-consuming and subjective than ECoG and fMRI, and entails unique risks. Above all, fMRI had most false positive face selective locations within 10 mm to the resected volumes and was susceptible to artifact at ventral temporal cortex which varies from the individual anatomical structures at temporal base. Therefore, broadband gamma mapping with ECoG turned out to be the most useful and reliable markers to identify face selective locations. Moreover, a multimodal approach including these two modalities confirmed co-localized face locations along anatomical face area in 3/5 patients. This outcome suggests that combined mapping results of co-localized face selective locations can improve specificity by minimizing false positives. Combined mappings may also further reduce the risks of ECS by minimizing the required locations for stimulation. All modalities should be considered in a clinical mapping protocol, and combined mapping results of co-localized face selective locations improve the specificity of ECoG and fMRI, and at the same time improve the sensitivity of ECS.

## Data Availability Statement

The datasets presented in this article are not readily available because the datasets include hospital patient data. Requests to access the datasets should be directed to TS, sanataka9103@gmail.com.

## Ethics Statement

The studies involving human participants were reviewed and approved by Asahikawa Medical University Research Ethics Committee. Written informed consent to participate in this study was provided by the participants’ legal guardian/next of kin.

## Author Contributions

TS: drafting of manuscript, acquisition and analysis of data, interpreting and validation of the results, and operations. CK: study conception and design, acquisition and analysis of data, interpreting and validation of the results, and supervision. MJ: design of paradigms and data processing, analysis of data, and visualization. JG: design of paradigms and data processing. HO, TM, and RA: acquisition of data and operations. CG: software development, supervision, and project administration. All authors contributed to the article and approved the submitted version.

## Conflict of Interest

CK, MJ, and JG are employees of g.tec medical engineering GmbH. CG is the CEO of g.tec medical engineering GmbH. The remaining authors declare that the research was conducted in the absence of any commercial or financial relationships that could be construed as a potential conflict of interest.

## References

[B1] Allison T. Ginter H. Mccarthy G. Nobre A. C. Puce A. Luby M. (1994a). Face recognition in human extrastriate cortex. *J. Neurophysiol.* 71 821–825. 10.1152/jn.1994.71.2.821 8176446

[B2] Allison T. McCarthy G. Nobre A. Puce A. Belger A. (1994b). Human extrastriate visual cortex and the perception of faces, words, numbers, and colors. *Cereb. Cortex* 4 544–554. 10.1093/cercor/4.5.544 7833655

[B3] Barton J. J. Press D. Z. Keenan J. P. O’connor M. (2002). Lesions of the fusiform face area impair perception of facial configuration in prosopagnosia. *Neurology* 58 71–78. 10.1212/wnl.58.1.71 11781408

[B4] Barton J. J. (2008). Structure and function in acquired prosopagnosia: lessons from a series of 10 patients with brain damage. *J. Neuropsychol.* 2 197–225. 10.1348/174866407x214172 19334311

[B5] Benton A. (1990). Facial recognition 1990. *Cortex* 26 491–499. 2081388 10.1016/s0010-9452(13)80299-7

[B6] Bodamer J. (1947). Die-Prosop-agnosie. Arch. Psychiatr. Nervenkrankh. 179, 6–54. English translation by Ellis, HD and Florence, M. (1990). *Cogn. Neuropsychol.* 7 81–105. 10.1080/02643299008253437

[B7] Bouvier S. E. Engel S. A. (2006). Behavioral deficits and cortical damage loci in cerebral achromatopsia. *Cereb. Cortex* 16 183–191. 10.1093/cercor/bhi096 15858161

[B8] Bukach C. M. Bub D. N. Gauthier I. Tarr M. J. (2006). Perceptual expertise effects are not all or none: spatially limited perceptual expertise for faces in a case of prosopagnosia. *J. Cogn. Neurosci.* 18 48–63. 10.1162/089892906775250094 16417682

[B9] Chong S. C. Jo S. Park K. M. Joo E. Y. Lee M.-J. Hong S. C. (2013). Interaction between the electrical stimulation of a face-selective area and the perception of face stimuli. *NeuroImage* 77 70–76. 10.1016/j.neuroimage.2013.01.074 23558104

[B10] Collin C. A. Therrien M. E. Campbell K. B. Hamm J. P. (2012). Effects of band-pass spatial frequency filtering of face and object images on the amplitude of N170. *Perception* 41 717–732. 10.1068/p7056 23094460

[B11] Corrivetti F. Herbet G. Moritz-Gasser S. Duffau H. (2017). Prosopagnosia induced by a left anterior temporal lobectomy following a right temporo-occipital resection in a multicentric diffuse low-grade glioma. *World Neurosurg*. 97:756.e1–756.e5. 10.1016/j.wneu.2016.10.025 27756661

[B12] Dale A. M. Fischl B. Sereno M. I. (1999). Cortical surface-based analysis: I. Segmentation and surface reconstruction. *NeuroImage* 9 179–194. 10.1006/nimg.1998.0395 9931268

[B13] Damasio A. R. Damasio H. Van Hoesen G. W. (1982). Prosopagnosia: anatomic basis and behavioral mechanisms. *Neurology* 32 331–331. 10.1212/wnl.32.4.331 7199655

[B14] De Renzi E. (1986). “Current issues on prosopagnosia,” in *Aspects of face Processing*, eds Ellis H. D. Jeeves M. A. Newcombe F. Young A. (Dordrecht: Springer), 243–252. 10.1007/978-94-009-4420-6_26

[B15] De Renzi E. Faglioni P. Grossi D. Nichelli P. (1991). Apperceptive and associative forms of prosopagnosia. *Cortex* 27 213–221. 10.1016/s0010-9452(13)80125-6 1879150

[B16] Devlin J. T. Russell R. P. Davis M. H. Price C. J. Wilson J. Moss H. E. (2000). Susceptibility-induced loss of signal: comparing PET and fMRI on a semantic task. *NeuroImage* 11 589–600. 10.1006/nimg.2000.0595 10860788

[B17] Engell A. D. McCarthy G. (2011). The relationship of gamma oscillations and face-specific ERPs recorded subdurally from occipitotemporal cortex. *Cereb. Cortex* 21 1213–1221. 10.1093/cercor/bhq206 20961973 PMC3077434

[B18] Evans J. J. Heggs A. Antoun N. Hodges J. R. (1995). Progressive prosopagnosia associated with selective right temporal lobe atrophy: a new syndrome? *Brain* 118(Pt 1) 1–13. 10.1093/brain/118.1.1 7894996

[B19] Farmer H. Hewstone M. Spiegler O. Morse H. Saifullah A. Pan X. (2020). Positive intergroup contact modulates fusiform gyrus activity to black and white faces. *Sci. Rep.* 10 1–13. 10.1038/s41598-020-59633-9 32060333 PMC7021708

[B20] Gaglianese A. Vansteensel M. J. Harvey B. M. Dumoulin S. O. Petridou N. Ramsey N. F. (2017). Correspondence between fMRI and electrophysiology during visual motion processing in human MT+. *NeuroImage* 155 480–489. 10.1016/j.neuroimage.2017.04.007 28389384 PMC5511559

[B21] Gainotti G. (2013). Is the right anterior temporal variant of prosopagnosia a form of ‘associative prosopagnosia’or a form of ‘multimodal person recognition disorder’? *Neuropsychol. Rev.* 23 99–110. 10.1007/s11065-013-9232-7 23579426

[B22] Gainotti G. Barbier A. Marra C. (2003). Slowly progressive defect in recognition of familiar people in a patient with right anterior temporal atrophy. *Brain* 126 792–803. 10.1093/brain/awg092 12615639

[B23] Gauthier I. Tarr M. J. Moylan J. Skudlarski P. Gore J. C. Anderson A. W. (2000). The fusiform “face area” is part of a network that processes faces at the individual level. *J. Cogn. Neurosci.* 12 495–504. 10.1162/089892900562165 10931774

[B24] Ghuman A. S. Brunet N. M. Li Y. Konecky R. O. Pyles J. A. Walls S. A. (2014). Dynamic encoding of face information in the human fusiform gyrus. *Nat. Commun*. 5:5672. 10.1038/ncomms6672 25482825 PMC4339092

[B25] Golarai G. Liberman A. Grill-Spector K. (2017). Experience shapes the development of neural substrates of face processing in human ventral temporal cortex. *Cereb. Cortex* 27 1229–1244. 10.1093/cercor/bhv314 26683171 PMC6161183

[B26] Haglund M. M. Berger M. S. Shamseldin M. Lettich E. Ojemann G. A. (1994). Cortical localization of temporal lobe language sites in patients with gliomas. *Neurosurgery* 34 567–576. 10.1227/00006123-199404000-00001 7516498

[B27] Haufe S. DeGuzman P. Henin S. Arcaro M. Honey C. J. Hasson U. (2018). Elucidating relations between fMRI, ECoG, and EEG through a common natural stimulus. *NeuroImage* 179 79–91. 10.1016/j.neuroimage.2018.06.016 29902585 PMC6063527

[B28] Haxby J. V. Horwitz B. Ungerleider L. G. Maisog J. M. Pietrini P. Grady C. L. (1994). The functional organization of human extrastriate cortex: a PET-rCBF study of selective attention to faces and locations. *J. Neurosci*. 14 6336–6353. 10.1523/jneurosci.14-11-06336.1994 7965040 PMC6577268

[B29] Haxby J. V. Hoffman E. A. Gobbini M. I. (2000). The distributed human neural system for face perception. *Trends Cogn. Sci.* 4 223–233. 10.1016/s1364-6613(00)01482-0 10827445

[B30] Hermes D. Miller K. J. Vansteensel M. J. Aarnoutse E. J. Leijten F. S. Ramsey N. F. (2012). Neurophysiologic correlates of fMRI in human motor cortex. *Hum. Brain Mapp*. 33 1689–1699. 10.1002/hbm.21314 21692146 PMC6870225

[B31] Hoffman E. A. Haxby J. V. (2000). Distinct representations of eye gaze and identity in the distributed human neural system for face perception. *Nat. Neurosci.* 3 80–84. 10.1038/71152 10607399

[B32] Ishai A. (2008). Let’s face it: it’sa cortical network. *NeuroImage* 40 415–419. 10.1016/j.neuroimage.2007.10.040 18063389

[B33] Jacques C. Witthoft N. Weiner K. S. Foster B. L. Rangarajan V. Hermes D. (2016). Corresponding ECoG and fMRI category-selective signals in human ventral temporal cortex. *Neuropsychologia* 83 14–28. 10.1016/j.neuropsychologia.2015.07.024 26212070 PMC4724347

[B34] Jonas J. Descoins M. Koessler L. Colnat-Coulbois S. Sauvée M. Guye M. (2012). Focal electrical intracerebral stimulation of a face-sensitive area causes transient prosopagnosia. *Neuroscience* 222 281–288. 10.1016/j.neuroscience.2012.07.021 22813996

[B35] Jonas J. Jacques C. Liu-Shuang J. Brissart H. Colnat-Coulbois S. Maillard L. (2016). A face-selective ventral occipito-temporal map of the human brain with intracerebral potentials. *Proc. Natl. Acad. Sci.U.S.A.* 113 E4088–E4097. 10.1073/pnas.1522033113 27354526 PMC4948344

[B36] Jonas J. Rossion B. Brissart H. Frismand S. Jacques C. Hossu G. (2015). Beyond the core face-processing network: intracerebral stimulation of a face-selective area in the right anterior fusiform gyrus elicits transient prosopagnosia. *Cortex* 72 140–155. 10.1016/j.cortex.2015.05.026 26143305

[B37] Kanwisher N. McDermott J. Chun M. M. (1997). The fusiform face area: a module in human extrastriate cortex specialized for face perception. *J. Neurosci.* 17 4302–4311. 10.1523/jneurosci.17-11-04302.1997 9151747 PMC6573547

[B38] Klopp J. Halgren E. Marinkovic K. Nenov V. (1999). Face-selective spectral changes in the human fusiform gyrus. *Clin. Neurophysiol.* 110 676–682. 10.1016/s1388-2457(98)00039-x 10378737

[B39] Koch S. P. Werner P. Steinbrink J. Fries P. Obrig H. (2009). Stimulus-induced and state-dependent sustained gamma activity is tightly coupled to the hemodynamic response in humans. *J. Neurosci.* 29 13962–13970. 19890006 10.1523/JNEUROSCI.1402-09.2009 PMC6666720

[B40] Ku S.-P. Tolias A. S. Logothetis N. K. Goense J. (2011). fMRI of the face-processing network in the ventral temporal lobe of awake and anesthetized macaques. *Neuron* 70 352–362. 10.1016/j.neuron.2011.02.048 21521619

[B41] Lachaux J.-P. George N. Tallon-Baudry C. Martinerie J. Hugueville L. Minotti L. (2005). The many faces of the gamma band response to complex visual stimuli. *NeuroImage* 25 491–501. 10.1016/j.neuroimage.2004.11.052 15784428

[B42] Liu J. Tian J. Lee K. Li J. (2008). A study on neural mechanism of face processing based on fMRI. *Prog. Nat. Sci.* 18 201–207. 10.1016/j.pnsc.2007.06.006

[B43] Mattson A. J. Levin H. S. Grafman J. (2000). A case of prosopagnosia following moderate closed head injury with left hemisphere focal lesion. *Cortex* 36, 125–137. 10.1016/s0010-9452(08)70841-4 10728902

[B44] Meadows J. (1974). The anatomical basis of prosopagnosia. *J. Neurol. Neurosurg. Psychiatry* 37 489–501. 10.1136/jnnp.37.5.489 4209556 PMC494693

[B45] Mesad S. Laff R. Devinsky O. (2003). Transient postoperative prosopagnosia. *Epilepsy Behav*. 4 567–570. 10.1016/j.yebeh.2003.07.012 14527501

[B46] Mundel T. Milton J. G. Dimitrov A. Wilson H. W. Pelizzari C. Uftring S. (2003). Transient inability to distinguish between faces: electrophysiologic studies. *J. Clin. Neurophysiol.* 20 102–110. 10.1097/00004691-200304000-00003 12766682

[B47] Nasr S. Tootell R. B. (2012). Role of fusiform and anterior temporal cortical areas in facial recognition. *NeuroImage* 63 1743–1753. 10.1016/j.neuroimage.2012.08.031 23034518 PMC3472036

[B48] Ojemann G. Ojemann J. Lettich E. Berger M. (1989). Cortical language localization in left, dominant hemisphere: an electrical stimulation mapping investigation in 117 patients. *J. Neurosurg*. 71 316–326. 10.3171/jns/2008/108/2/0411 2769383

[B49] Ojemann J. G. Akbudak E. Snyder A. Z. McKinstry R. C. Raichle M. E. Conturo T. E. (1997). Anatomic localization and quantitative analysis of gradient refocused echo-planar fMRI susceptibility artifacts. *NeuroImage* 6 156–167. 10.1006/nimg.1997.0289 9344820

[B50] Ojemann G. A. Corina D. P. Corrigan N. Schoenfield-Mcneill J. Poliakov A. Zamora L. (2010). Neuronal correlates of functional magnetic resonance imaging in human temporal cortex. *Brain* 133 46–59. 10.1093/brain/awp227 19773355 PMC2801320

[B51] Parvizi J. Jacques C. Foster B. L. Withoft N. Rangarajan V. Weiner K. S. (2012). Electrical stimulation of human fusiform face-selective regions distorts face perception. *J. Neurosci.* 32 14915–14920. 10.1523/jneurosci.2609-12.2012 23100414 PMC3517886

[B52] Penfield W. Boldrey E. (1937). Somatic motor and sensory representation in the cerebral cortex of man as studied by electrical stimulation. *Brain* 60 389–443. 10.1192/bjp.84.352.868-a

[B53] Penny W. D. Friston K. J. Ashburner J. T. Kiebel S. J. Nichols T. E. (2011). *Statistical Parametric Mapping: The Analysis of Functional Brain Images.* Burlington, MA: Elsevier.

[B54] Pfaender M. D’Souza W. Trost N. Litewka L. Paine M. Cook M. (2004). Visual disturbances representing occipital lobe epilepsy in patients with cerebral calcifications and coeliac disease: a case series. *J. Neurol. Neurosurg. Psychiatry* 75 1623–1625. 10.1136/jnnp.2003.031229 15489401 PMC1738780

[B55] Pitcher D. Walsh V. Duchaine B. (2011). The role of the occipital face area in the cortical face perception network. *Exp. Brain Res.* 209 481–493. 10.1007/s00221-011-2579-1 21318346

[B56] Pitcher D. Walsh V. Yovel G. Duchaine B. (2007). TMS evidence for the involvement of the right occipital face area in early face processing. *Curr. Biol.* 17 1568–1573. 10.1016/j.cub.2007.07.063 17764942

[B57] Puce A. Allison T. Gore J. C. McCarthy G. (1995). Face-sensitive regions in human extrastriate cortex studied by functional MRI. *J. Neurophysiol*. 74 1192–1199. 10.1152/jn.1995.74.3.1192 7500143

[B58] Puce A. Allison T. Asgari M. Gore J. C. Mccarthy G. (1996). Differential sensitivity of human visual cortex to faces, letterstrings, and textures: a functional magnetic resonance imaging study. *J. Neurosci.* 16 5205–5215. 10.1523/JNEUROSCI.16-16-05205.1996 8756449 PMC6579313

[B59] Puce A. Allison T. Bentin S. Gore J. C. Mccarthy G. (1998). Temporal cortex activation in humans viewing eye and mouth movements. *J. Neurosci.* 18 2188–2199. 10.1523/JNEUROSCI.18-06-02188.1998 9482803 PMC6792917

[B60] Puce A. Allison T. Spencer S. S. Spencer D. D. Mccarthy G. (1997). Comparison of cortical activation evoked by faces measured by intracranial field potentials and functional MRI: two case studies. *Hum. Brain Mapp.* 5 298–305. 10.1002/(SICI)1097-019319975:4<298::AID-HBM16<3.0.CO;2-A 20408232

[B61] Rangarajan V. Hermes D. Foster B. L. Weiner K. S. Jacques C. Grill-Spector K. (2014). Electrical stimulation of the left and right human fusiform gyrus causes different effects in conscious face perception. *J. Neurosci.* 34 12828–12836. 10.1523/jneurosci.0527-14.2014 25232118 PMC4166163

[B62] Romero J. R. Ramirez D. M. Aglio L. S. Gugino L. D. (2011). Brain mapping using transcranial magnetic stimulation. *Neurosurg. Clin*. 22 141–152. 10.1016/j.nec.2010.11.002 21435567

[B63] Rossion B. Caldara R. Seghier M. Schuller A. M. Lazeyras F. Mayer E. (2003). A network of occipito-temporal face-sensitive areas besides the right middle fusiform gyrus is necessary for normal face processing. *Brain* 126 2381–2395. 10.1093/brain/awg241 12876150

[B64] Rossion B. Jacques C. Jonas J. (2018). Mapping face categorization in the human ventral occipitotemporal cortex with direct neural intracranial recordings. *Ann. N. Y. Acad. Sci.* 1426 5–24. 10.1111/nyas.13596 29479704

[B65] Sagar S. Rick J. Chandra A. Yagnik G. Aghi M. K. (2019). Functional brain mapping: overview of techniques and their application to neurosurgery. *Neurosurg. Rev.* 42 639–647. 10.1007/s10143-018-1007-4 30006663

[B66] Sanai N. Mirzadeh Z. Berger M. S. (2008). Functional outcome after language mapping for glioma resection. *N. Engl. J. Med.* 358 18–27. 10.1056/nejmoa067819 18172171

[B67] Schalk G. Kapeller C. Guger C. Ogawa H. Hiroshima S. Lafer-Sousa R. (2017). Facephenes and rainbows: causal evidence for functional and anatomical specificity of face and color processing in the human brain. *Proc. Natl. Acad. Sci.U.S.A*. 114 12285–12290. 10.1073/pnas.1713447114 29087337 PMC5699078

[B68] Scheeringa R. Fries P. Petersson K.-M. Oostenveld R. Grothe I. Norris D. G. (2011). Neuronal dynamics underlying high-and low-frequency EEG oscillations contribute independently to the human BOLD signal. *Neuron* 69 572–583. 10.1016/j.neuron.2010.11.044 21315266

[B69] Scheffer I. E. Berkovic S. Capovilla G. Connolly M. B. French J. Guilhoto L. (2017). ILAE classification of the epilepsies: position paper of the ILAE commission for classification and terminology. *Epilepsia* 58 512–521. 10.1111/epi.13709 28276062 PMC5386840

[B70] Schwarz L. Kreifelts B. Wildgruber D. Erb M. Scheffler K. Ethofer T. (2019). Properties of face localizer activations and their application in functional magnetic resonance imaging (fMRI) fingerprinting. *PLoS One* 14:e0214997. 10.1371/journal.pone.0214997 31013276 PMC6478291

[B71] Sergent J. Ohta S. Macdonald B. (1992). Functional neuroanatomy of face and object processing: a positron emission tomography study. *Brain* 115 15–36. 10.1093/brain/115.1.15 1559150

[B72] Sollmann N. Fuss-Ruppenthal S. Zimmer C. Meyer B. Krieg S. M. (2018). Investigating stimulation protocols for language mapping by repetitive navigated transcranial magnetic stimulation. *Front. Behav. Neurosci.* 12:197. 10.3389/fnbeh.2018.00197 30250427 PMC6139335

[B73] Sorger B. Goebel R. Schiltz C. Rossion B. (2007). Understanding the functional neuroanatomy of acquired prosopagnosia. *NeuroImage* 35 836–852. 10.1016/j.neuroimage.2006.09.051 17303440

[B74] Swift J. Coon W. Guger C. Brunner P. Bunch M. Lynch T. (2018). Passive functional mapping of receptive language areas using electrocorticographic signals. *Clin. Neurophysiol.* 129 2517–2524. 10.1016/j.clinph.2018.09.007 30342252 PMC6414063

[B75] Takahashi N. Kawamura M. Hirayama K. Shiota J.-I. Isono O. (1995). Prosopagnosia: a clinical and anatomical study of four patients. *Cortex* 31 317–329. 10.1016/s0010-9452(13)80365-6 7555009

[B76] Tanji K. Iwasaki M. Nakasato N. Suzuki K. (2012). Face specific broadband electrocorticographic spectral power change in the rhinal cortex. *Neurosci. Lett*. 515 66–70. 10.1016/j.neulet.2012.03.020 22450049

[B77] Taylor I. Scheffer I. E. Berkovic S. F. (2003). Occipital epilepsies: identification of specific and newly recognized syndromes. *Brain* 126 753–769. 10.1093/brain/awg080 12615636

[B78] Tharin S. Golby A. (2007). Functional brain mapping and its applications to neurosurgery. *Neurosurgery* 60(4 Suppl 2) 185–201; discussion 201–182. 17415154 10.1227/01.NEU.0000255386.95464.52

[B79] Theodore W. H. (2003). Transcranial magnetic stimulation in epilepsy. *Epilepsy Curr.* 3 191–197. 10.1046/j.1535-7597.2003.03607.x 15346149 PMC321221

[B80] Wada Y. Yamamoto T. (2001). Selective impairment of facial recognition due to a haematoma restricted to the right fusiform and lateral occipital region. *J. Neurol. Neurosurg. Psychiatry* 71 254–257. 10.1136/jnnp.71.2.254 11459906 PMC1737516

[B81] Weiner K. S. Grill-Spector K. (2010). Sparsely-distributed organization of face and limb activations in human ventral temporal cortex. *NeuroImage* 52 1559–1573. 10.1016/j.neuroimage.2010.04.262 20457261 PMC3122128

[B82] Weiner K. S. Grill-Spector K. (2013). Neural representations of faces and limbs neighbor in human high-level visual cortex: evidence for a new organization principle. *Psychol. Res.* 77 74–97. 10.1007/s00426-011-0392-x 22139022 PMC3535411

[B83] Wen J. Yu T. Li Y. Li X. (2017). Using electrocorticography for presurgical language mapping in epilepsy patients. *J. Clin. Neurosci.* 44 320–322. 10.1016/j.jocn.2017.06.015 28676308

[B84] Winston J. S. Henson R. Fine-Goulden M. R. Dolan R. J. (2004). fMRI-adaptation reveals dissociable neural representations of identity and expression in face perception. *J. Neurophysiol.* 92 1830–1839. 10.1152/jn.00155.2004 15115795

